# Leonard William Sedgwick

**Published:** 1911-04-01

**Authors:** 


Leonard William Sedgwick, who died in London recently, aged eighty-two, had retired for some time from practice. He graduated M.D. of St. Andrews in 1859, after having become M.R.C.S. and L.S.A. in 1850. He studied at St. Thomas's Hospital and had been a vice-president of the Medical Society of London. His publications included a "Report on the Parasitic Theory of Disease"; "Mineral Waters of Tarasp"; and, with Mr. Power, a "Lexicon of Medicine."

